# Electrospray ionization mass spectrometry ion suppression/enhancement caused by column bleed for three mixed‐mode reversed‐phase/anion‐exchange high‐performance liquid chromatography columns

**DOI:** 10.1002/rcm.9098

**Published:** 2021-05-04

**Authors:** Thomas H. Walter, Melvin Blaze M. T., Cheryl Boissel

**Affiliations:** ^1^ Waters Corporation 34 Maple Street Milford MA USA

## Abstract

**Rationale:**

Mixed‐mode reversed‐phase/anion exchange liquid chromatography is useful for separations of mixtures containing anions (e.g. ionized acids). However, when using this form of liquid chromatography with mass spectrometry detection, the bleed of amine‐containing hydrolysis products from the columns may cause ion suppression or enhancement.

**Methods:**

Using electrospray ionization tandem quadrupole mass spectrometry detection, we determined the ion suppression or enhancement caused by column bleed for three mixed‐mode reversed‐phase/weak anion‐exchange columns containing stationary phases that differ in chemical structure. Two of the stationary phases are based on silica particles, while the third uses ethylene‐bridged hybrid organic/inorganic particles, which have improved hydrolytic stability. Mixtures of acidic and basic analytes were combined with the chromatography flow postcolumn, both with and without a column, and their mass spectrometry ion signal responses (peak areas) were determined. The ratio of signal response with and without a column is the matrix factor. Positive ion electrospray measurements were carried out using 0.1% formic acid (pH ~ 2.7) as a mobile phase additive, and 10mM ammonium formate (pH ~ 6.4) was used for negative ion electrospray detection.

**Results:**

The matrix factors under both positive and negative ionization modes were closest to 1 (0.74–1.16) for the hybrid particle‐based columns, showing minimal ion suppression or enhancement. In contrast, the silica‐based columns gave matrix factors ranging from 0.04 to 1.86, indicating high levels of ion suppression or enhancement. These results may be explained by the differences in the structures of the stationary phases, which affect the relative amounts of hydrolysis products that elute from the columns.

**Conclusions:**

The low levels of mass spectrometry ion suppression or enhancement caused by column bleed from the hybrid particle‐based columns should allow for accurate quantitative mass spectrometric detection combined with mixed‐mode reversed‐phase/weak anion‐exchange chromatography.

## 1 INTRODUCTION

Mixed‐mode reversed‐phase (RP)/anion‐exchange (AX) columns, which contain stationary phases having both hydrophobic and AX functionalities, provide a means to retain and separate anions (e.g. ionized acids) under RP conditions.^1^, ^2^, ^3^, ^4^, ^5^, ^6^ These columns have been most commonly used with optical detection, for example UV absorbance. When considering their use with electrospray ionization mass spectrometry (ESI‐MS), the hydrolytic stability of the stationary phase is an area of concern. Many mixed‐mode RP/AX stationary phases, particularly those based on silane‐bonded silica, suffer from relatively poor hydrolytic stability. This is reflected in the limited recommended pH ranges for these columns. For example, one brand of RP/AX columns is recommended for use in the pH range 1.5–4,^7^ while a second has a range of 2.5–7.5.^8^ Organic polymer‐based mixed‐mode columns have been reported to be stable over a wide pH range (e.g. 1–14), but suffer from low efficiencies.^9^ In recent work to improve the hydrolytic stability of silica‐based mixed‐mode RP/AX materials, a polymer‐coating approach was used.^10^ The resulting stationary phase was shown to have good stability when used with a pH 5 mobile phase at 60°C. To improve both acid and base stability without sacrificing column efficiency, a mixed‐mode RP/AX stationary phase based on ethylene‐bridged hybrid organic/inorganic particles^11^ was recently developed. This stationary phase has been shown to be stable from pH 2 to 10.^12^

Ion suppression or enhancement is an important concern in quantitative liquid chromatography (LC) ESI‐MS.^13^, ^14^, ^15^, ^16^ The primary cause is linked to components that co‐elute with the analytes and affect the ionization process. These components may originate from the sample, from mobile phase additives or from contaminants introduced during sample preparation.^13^, ^15^, ^16^ Hydrolysis products eluted from the column stationary phase, referred to as column bleed, are another potential source. Under acidic conditions, silane bonded phases hydrolyze to form organosilanols, which may condense to form organosiloxanes.^17^ Under basic conditions, not only is the bonded phase susceptible to hydrolysis, but the underlying silica particles may begin to be attacked, forming soluble silicates.^18^, ^19^ The stability of different bonded phases varies over a wide range, depending on the attachment chemistry, the structure and surface concentration of the bonded organic groups, and the chemical and physical properties of the underlying particles.^20^ Elution of bonded‐phase hydrolysis products containing amine groups from mixed‐mode RP/AX columns has been shown to cause problems due to their strong MS response. These problems include a high background signal in total ion current mode^21^ and low ion transmission in high‐resolution MS.^22^

Here, we compare the magnitude of ion suppression or enhancement due to column bleed for hybrid particle‐based mixed‐mode columns relative to two silica‐based mixed‐mode RP/AX columns. These three stationary phases employ different bonding chemistries as well as different particles. We describe these differences. The column bleed profiles of the three materials were determined using both an acidic mobile phase with positive ionization ESI (ESI+) and a neutral mobile phase with negative ionization ESI (ESI−). To quantify ion suppression or enhancement caused by hydrolysis products eluted from these columns, we monitored seven small‐molecule analytes under ESI+ and five small‐molecule analytes under ESI− using analyte‐specific selected reaction monitoring (SRM) transitions. The analyte mixtures were combined with the LC mobile phase flow postcolumn, both in the presence and in the absence of a column (using a union instead of a column). The ratio of signal response with and without a column is the matrix factor.

## 2 METHODS

### 2.1 Chemicals

LC‐MS grade acetonitrile was purchased from Honeywell (Muskegon, MI) and MS grade formic acid (FA) was purchased from Fisher Scientific (Hampton, NH). Ammonium formate and all analytes were purchased from Millipore‐Sigma (Burlington, MA). Deionized water was produced using a Millipore Milli‐Q system.

### 2.2 Sample and mobile phase preparation

The sample for the ESI+ test contained 10 μg/mL thiourea, 20 μg/mL thymine, 10 μg/mL adenine, 2 μg/mL 5‐fluorocytosine, 5 μg/mL thiamine, 15 μg/mL tryptophan and 1.5 μg/mL niflumic acid in 1:1 acetonitrile/0.1% (v/v) FA in water. For the ESI− test, the sample contained 10 μg/mL citric acid, 10 μg/mL malic acid, 10 μg/mL guanosine‐5′‐monophosphate disodium salt hydrate, 10 μg/mL thymidine‐5′‐monophosphate disodium salt hydrate and 1.5 μg/mL niflumic acid in 1:1 acetonitrile/0.1% (v/v) FA in water. The aqueous and organic mobile phases for the ESI+ test were 0.1% (v/v) FA in water and in acetonitrile, respectively, and for the ESI− test, the aqueous and organic mobile phase were 10mM ammonium formate in water and acetonitrile, respectively.

### 2.3 Instrumentation and columns

All analyses were performed using two ACQUITY UPLC H‐Class systems coupled to Xevo TQD mass spectrometers (Waters Corporation, Milford, MA): one for ESI+ tests and the other for ESI− tests. The IntelliStart fluidics system built into the mass spectrometers was used to infuse the analyte solution by combining with the LC flow postcolumn into the MS probe. Both UPLC systems were equipped with a quaternary solvent manager, a flow‐through needle sample manager and a column manager. Data acquisition and analysis were performed using MassLynx V4.2. Atlantis PREMIER BEH C_18_ AX columns (column W) (1.7 μm, 2.5 μm and 5 μm, 2.1 × 150 mm) were also obtained from Waters Corporation (Milford, MA). Primesep B columns (column P) (5 μm, 2.1 × 150 mm) were obtained from SiELC Technologies (Wheeling, IL) and Acclaim Mixed‐Mode WAX‐1 columns (column A) (5 μm, 2.1 × 150 mm) were purchased from Thermo Fisher Scientific (Waltham, MA).

### 2.4 Experimental plan

**FIGURE 1:**
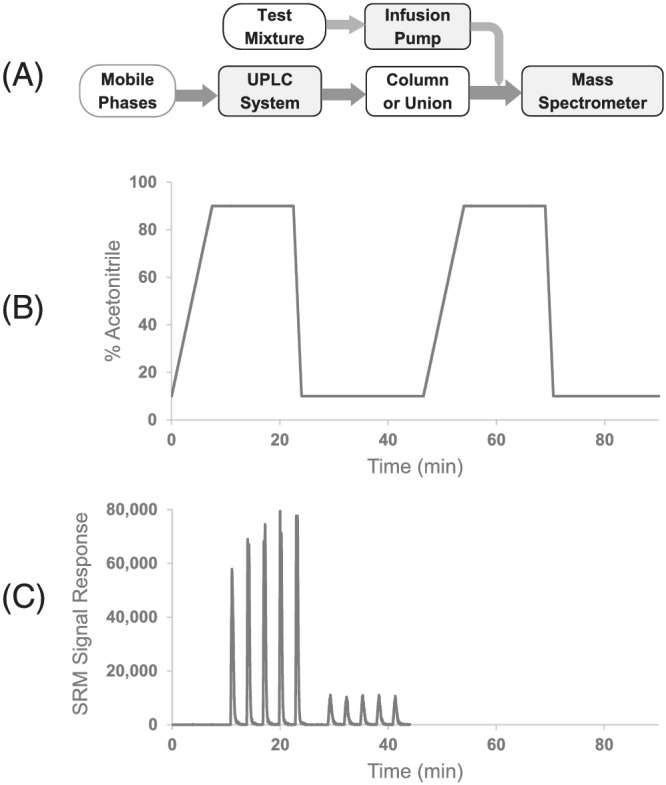
(A) Schematic of the instrument setup for the matrix factor study. (B) Chart showing the mobile phase double gradient (% acetonitrile versus time). (C) Representative SRM signal response showing the timing of the postcolumn infusions relative to the mobile phase gradient. The second portion of the gradient (43–93 min) was used to record mass spectra from *m*/*z* 50 to 1000

To evaluate ESI‐MS signal suppression or enhancement due to column stationary phase bleed, seven different analytes under ESI+ and five different analytes under ESI− were monitored using analyte‐specific SRM transitions. The analytes chosen are readily detectable under ESI+ and/or ESI− modes of ionization and include acidic, basic and neutral analytes with molecular weights ranging from 76 to 363 Da. The LC and MS conditions are given in Tables 1 and 2. The analyte mixtures were infused postcolumn into the LC mobile phase flow before entering the ESI‐MS setup, as depicted in Figure 1A. A mobile phase gradient was carried out, with the acetonitrile content increasing from 10% to 90%. A double gradient was executed five times for each column, and the results for the fifth run were used for the calculations. The infusions of the analyte mixtures were performed at 2.5 min intervals at a flow rate of 200 μL/min for 0.5 min while the acetonitrile content was held at 90% and 10% (see Figures 1B and 1C). For the ESI+ measurements, five replicate infusions were performed during both the 10% and 90% organic portions of the gradient, and the results for the second through the fifth infusions were used in the calculations. For the ESI− measurements, five replicate infusions were performed during the 90% organic portion of the gradient, and four infusions during the 10% organic portion, and the results for the third through the fifth (90% organic) and second through the fourth infusions (10% organic) were used in the calculations. During the second portion of the gradient (from 43.0 to 93.0 min), full‐scan mass spectra (*m*/*z* 50–1000) of the column bleed were recorded during the 90% and 10% acetonitrile portions of the gradient. The ESI‐MS signal responses for the postcolumn‐infused analytes were measured in the presence and absence of the column for three W columns (1.7 μm, 2.1 × 50 mm; 2.5 μm, 2.1 × 50 mm; and 5 μm, 2.1 × 150 mm), one P column (5 μm, 2.1 × 150 mm) and one A column (3 μm, 2.1 × 150 mm). Separate columns were used for ESI+ and ESI− experiments. The matrix factors imparted by the column stationary phase bleed on these analytes were calculated using the following equation for each individual measurement:1



$$Matrix\;Factor\;(MF)\;=\;\frac{Analyte\;peak\;area\;in\;the\;presence\;of\;column}{Analyte\;peak\;area\;in\;the\;absence\;of\;column}$$

**TABLE 1:** LC conditions

Mobile phase A (ESI+)	0.1% (v/v) formic acid in water		
Mobile phase B (ESI+)	0.1% (v/v) formic acid in acetonitrile		
Mobile phase A (ESI−)	10mM ammonium formate in water		
Mobile phase B (ESI−)	Acetonitrile		
Flow rate:	0.2 mL/min		
Column temperature	50°C		
Time (min)	%A	%B	Curve
Initial	90	10	Initial
7.5	10	90	6
22.5	10	90	6
24.0	90	10	6
46.5	90	10	6
54.0	10	90	6
69.0	10	90	6
70.5	90	10	6
93.0	90	10	6
96.0	90	10	6

**TABLE 2:** MS conditions

Source conditions			
Capillary voltage (kV)	ESI+, 2.90; ESI−, 2.0		
Cone voltage (V)	45		
Desolvation temperature	250°C		
Desolvation gas (L/h)	600		

SRM transitions, ESI+			
Compound name	Precursor ion (*m*/*z*)	Product ion (*m*/*z*)	Collision energy (V)
Thiourea	76.91	43.05	80
Thymine	126.94	53.11	26
5‐Fluorocytosine	129.97	57.95	32
Adenine	136.02	93.99	46
Tryptophan	205.06	145.99	20
Thiamine	265.07	121.95	16
Niflumic acid	282.92	144.96	40

SRM transitions, ESI−			
Compound name	Precursor ion (*m*/*z*)	Product ion (*m*/*z*)	Collision energy (V)
Malic acid	133.00	71.00	14
Citric acid	191.10	111.00	14
Niflumic acid	281.10	237.20	35
Thymidine‐5′‐monophosphate	321.10	194.90	22
Guanosine‐5′‐monophosphate	362.10	78.90	28

## 3 RESULTS AND DISCUSSION

### 3.1 Chemical and physical properties of the three stationary phases

**FIGURE 2:**
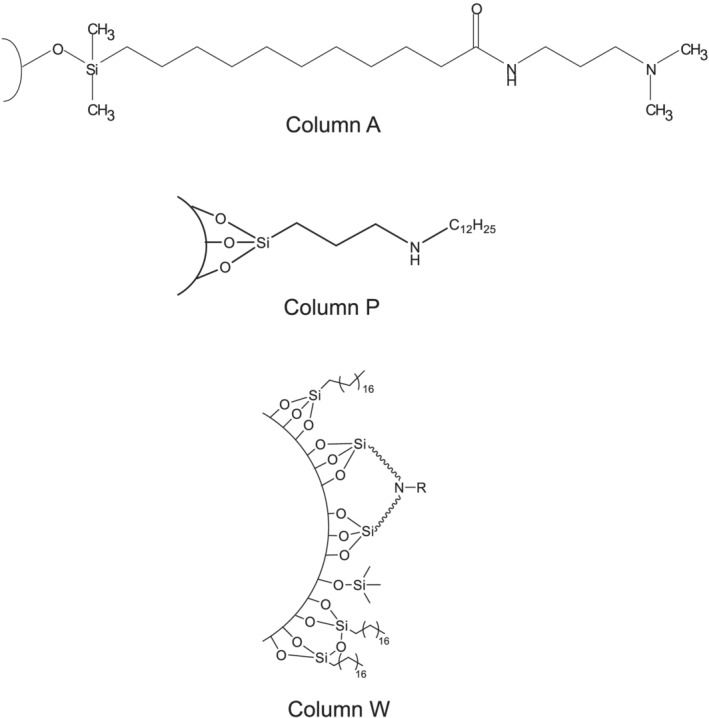
Schematic representations of bonded phases for columns A, P and W

The properties of the three stationary phases are summarized and compared in Table 3. The stationary phase of column W is based on ethylene‐bridged hybrid particles and contains both C_18_ groups and tertiary alkylamine moieties, the latter creating a positive surface charge below approximately pH 8–9.^12^, ^23^ This stationary phase is endcapped to reduce the concentration of residual silanols and to improve the stability of the stationary phase to basic mobile phases. The stationary phases for columns P and A are based on silica particles. The structures of the bonded phases for these columns have previously been reported^1^ and are shown in Figure 2. The surface concentrations of these bonded phases were calculated based on their carbon contents and surface areas.^24^ The nature of the attachment to the silica surface for these bonded phases was established using ^29^Si cross‐polarization magic angle spinning NMR spectroscopy.^25^, ^26^ The results indicate that the stationary phase of column A is monofunctionally bonded, while that of column P is trifunctionally bonded. Unlike column W, the stationary phases for columns A and P contain bonded groups that incorporate both hydrophobic and AX moieties together in the same structure. In contrast, the stationary phase of column W is bonded with separate hydrophobic and AX groups, with the AX group surface concentration being approximately five times lower than that of the C_18_ groups.

**TABLE 3:** Chemical and physical properties of stationary phases evaluated

	Particle properties				Bonded‐phase properties		
Column	Material	Pore size (Å)	Pore volume (cm^3^/g)	Surface area (m^2^/g)	%C	Surface concentration of primary group (μmol/m^2^)	Endcapping
W	Ethylene‐bridged hybrid	95	0.7	270	16.83	1.6	Yes
P	Silica	100	0.9	300	6.96	1.4	No
A	Silica	120	0.9	300	13.47	2.6	Unknown

### 3.2 Effect of column bleed on ESI+ MS signal response

**FIGURE 3:**
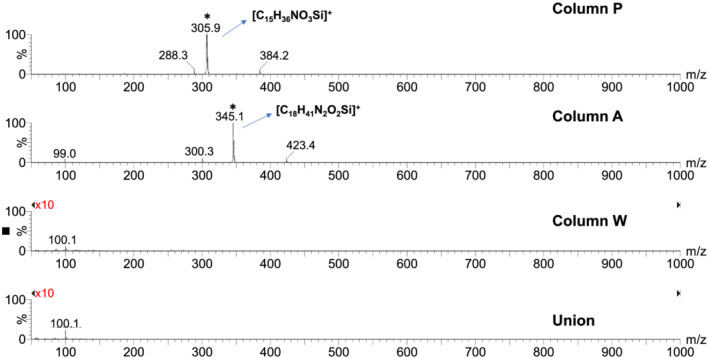
Representative ESI+ mass spectra of column bleed for the three columns and the union at a mobile phase composition of 10:90 acetonitrile/0.1% (v/v) FA in water. The major peaks are labelled with the proposed elemental formulas. Peaks that saturated the MS detector are marked with asterisks. The vertical scales were magnified tenfold for the bottom two mass spectra

**FIGURE 4:**
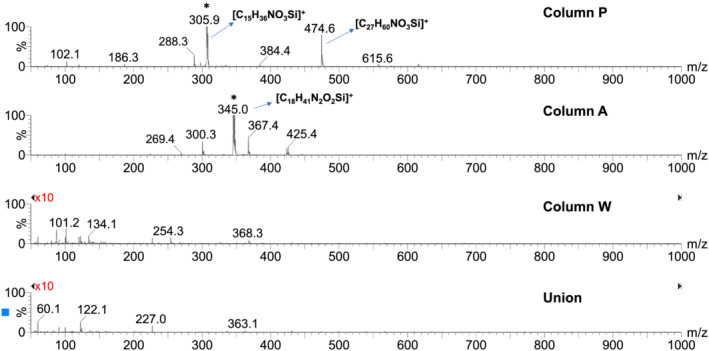
Representative ESI+ mass spectra of column bleed for the three columns and the union at a mobile phase composition of 90:10 acetonitrile/0.1% (v/v) FA in water. The major peaks are labelled with the proposed elemental formulas. Peaks that saturated the MS detector are marked with asterisks. The vertical scales were magnified tenfold for the bottom two mass spectra

Elution of bonded phase hydrolysis products from columns can affect the signal intensity for co‐eluting analytes when using ESI‐MS. Bonded‐phase hydrolysis products containing ionizable groups, such as amines, are particularly problematic because of their strong MS response. Full‐scan ESI+ mass spectra (*m*/*z* 50–1000) obtained using 0.1% FA (pH ~ 2.7) as a mobile phase additive with columns A and P showed very intense MS peaks that are attributed to bonded‐phase hydrolysis products (see Figures 3 and 4). The mass spectra obtained at a mobile phase composition of 10/90 acetonitrile/aqueous 0.1% FA are shown in Figure 3, while the mass spectra in Figure 4 were recorded at a composition of 90/10 acetonitrile/aqueous 0.1% FA. For column P, for both mobile phase compositions a strong peak saturating the detector was observed at *m*/*z* 305.9. This is consistent with the protonated form of the trisilanol hydrolysis product expected to be formed from the bonded‐phase structure shown in Figure 2, with an elemental formula of [C_15_H_36_NO_3_Si]^+^. The less intense peak at *m*/*z* 474.6 observed in 90/10 acetonitrile/aqueous 0.1% FA (Figure 4) is tentatively ascribed to the protonated form of a related bonded‐phase hydrolysis product having two dodecyl groups attached to the nitrogen atom, with an elemental formula of [C_27_H_60_NO_3_Si]^+^. This appears plausible considering the synthetic chemistry believed to be used to make the stationary phase of column P.^27^ For column A, for both mobile phase compositions an intense peak saturating the detector was observed at *m*/*z* 345.1. This is attributed to the protonated form of the silanol hydrolysis product expected to be formed from the structure shown in Figure 2, with an elemental formula of [C_18_H_41_N_2_O_2_Si]^+^. Column W showed a few peaks with intensities 25–35 times lower than those of the other two columns. This is most evident in the mass spectrum observed in 90/10 acetonitrile/aqueous 0.1% FA (see Figure 4).

**FIGURE 5:**
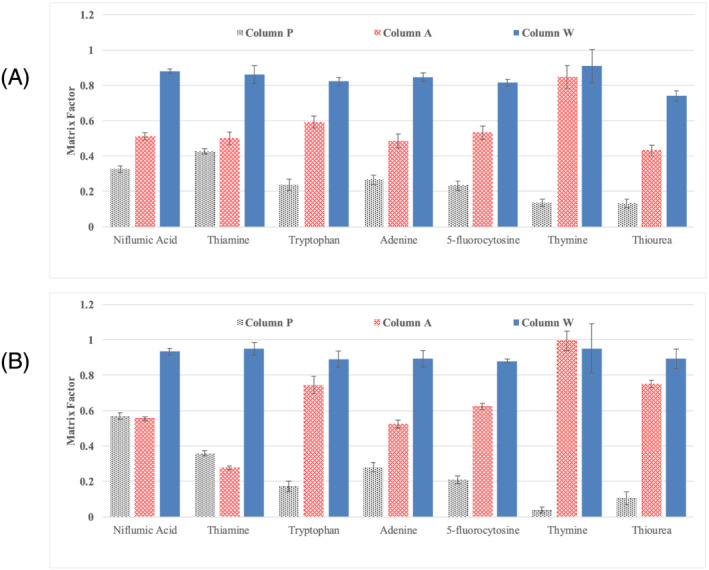
Comparison of matrix factors from column bleed for column P (black bars with vertical lines), column A (red bars with horizontal lines) and column W (blue solid bars). The error bars show one standard deviation for five measurements each on one column A and one column P and fifteen measurements on three W columns. (A) 0.1% FA mobile phase additive and ESI+ detection at 10% acetonitrile; (B) 0.1% FA mobile phase additive and ESI+ detection at 90% acetonitrile

Figure 5 shows a comparison of the matrix factors for the representative analytes measured under ESI+ with 0.1% FA mobile phase at 90% acetonitrile (Figure 5A) and at 10% acetonitrile (Figure 5B) for the three columns. A matrix factor value of 1 indicates the signal responses in the presence and the absence of the column are the same, signifying no matrix effect. A matrix factor value less than 1 indicates that the MS signal response in the presence of the column is lower than that in the absence of the column, signifying suppression of the analyte MS signal response primarily caused by column bleed. A matrix factor greater than 1 indicates the MS signal response in the presence of the column is greater than in the absence of column, signifying enhancement in the analyte MS signal response primarily caused by column bleed. Columns P and A (black and red bars, respectively) exhibited significant signal suppression for many of the analytes, as shown by matrix factors below 0.6. This was the case at both 10% and 90% acetonitrile. In contrast, column W (blue bars) showed matrix factors ranging from 0.74 to 0.95, indicating little signal suppression. The results for the latter column represent the grand average of the values obtained using one 1.7 μm, one 2.5 μm and one 5 μm column with five replicate measurements for each column, and the error bars show ± one pooled standard deviation. The results for columns A and P represent the average of five measurements each and the error bars show ± one standard deviation. We attribute the lower matrix factors for column W to a lower concentration of amine‐containing bonded‐phase hydrolysis products.

### 3.3 Effect of column bleed on ESI− MS signal response

**FIGURE 6:**
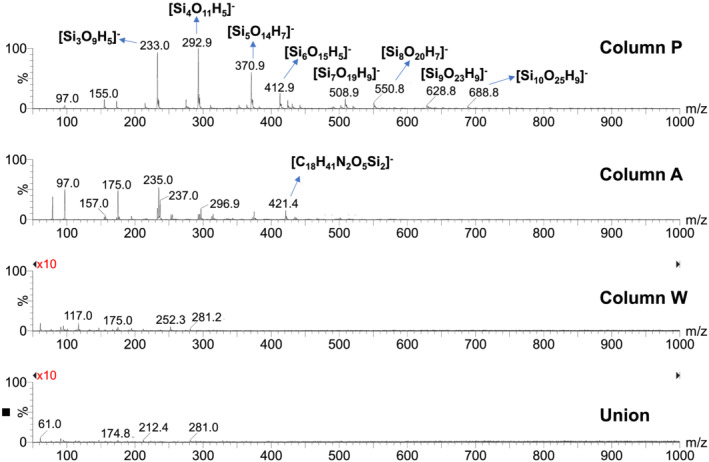
Representative ESI− mass spectra of column bleed for the three columns and the union at a mobile phase composition of 10:90 acetonitrile/10mM ammonium formate in water. The major peaks are labelled with the proposed elemental formulas. The vertical scales were magnified tenfold for the bottom two mass spectra

**FIGURE 7:**
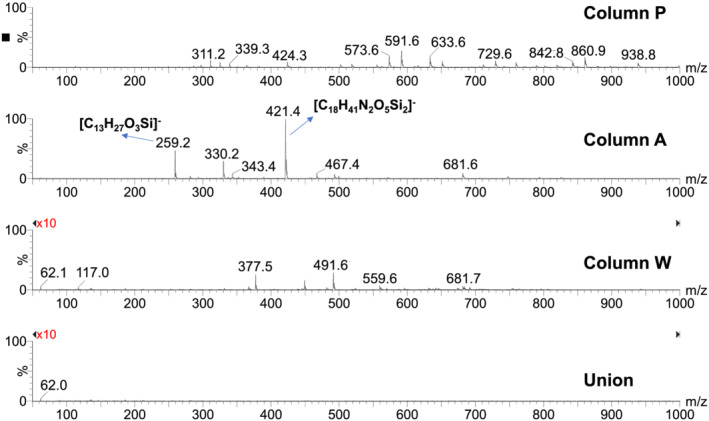
Representative ESI− mass spectra of column bleed for the three columns and the union at a mobile phase composition of 90:10 acetonitrile/10mM ammonium formate in water. The major peaks are labelled with the proposed elemental formulas. The vertical scales were magnified tenfold for the bottom two mass spectra

In the ESI− experiments performed using a 10mM ammonium formate (pH ~ 6.4) mobile phase, the mass spectra of the column bleed showed a number of moderate intensity peaks. The mass spectrum obtained for column P at the 10/90 acetonitrile/aqueous mobile phase composition (Figure 6) shows a series of peaks that match the known *m*/*z* values of silicate oligomers containing from three to ten silicon atoms.^28^ These likely arise from hydrolysis of the silica particles. The mobile phase pH of approximately 6.4 used for this experiment was above the recommended limit for column P. The mass spectrum observed for column A at the same mobile phase composition does not show these peaks, but one peak has a *m*/*z* value (421.4) consistent with a bonded‐phase hydrolysis product bonded to a silicate monomer, with an elemental formula of [C_18_H_41_N_2_O_5_Si_2_]^−^ (Figure 6). This peak is more intense in the 90/10 acetonitrile/aqueous composition, and a peak at *m*/*z* 259.2 is observed that is consistent with a bonded‐phase hydrolysis product where the amide group has been hydrolyzed to form a carboxylate group, giving a species with an elemental formula of [C_13_H_27_O_3_Si]^−^ (Figure 7). Column W showed only a few peaks with intensities more than 10 times lower than those of the other two columns.

**FIGURE 8:**
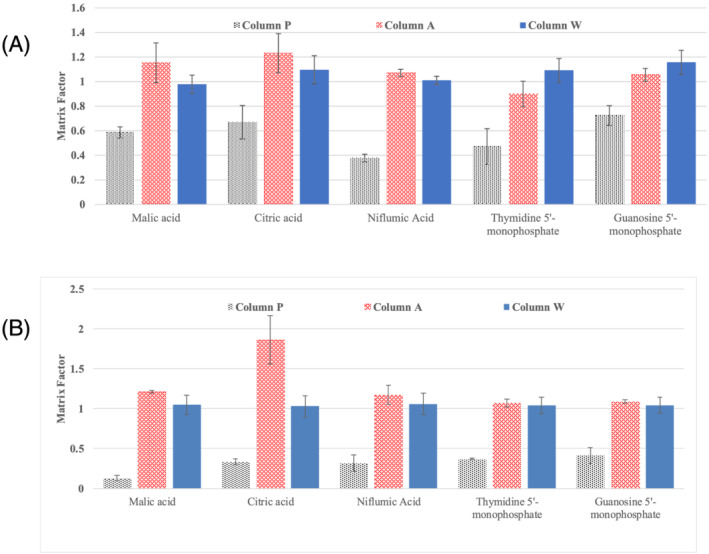
Comparison of matrix factors from column bleed for column P (black bars with vertical lines), column A (red bars with horizontal lines) and column W (blue solid bars). The error bars show one standard deviation for four (A) or five (B) measurements each on one column A and one column P and twelve (A) or fifteen (B) measurements on three W columns. (A) 10mM ammonium formate and ESI− detection at 10% acetonitrile; (B) 10mM ammonium formate and ESI− detection at 90% acetonitrile

Shown in Figure 8 is a comparison of the matrix factors for the three columns for the representative analytes measured under ESI− with 10mM ammonium formate mobile phase additive at 10% acetonitrile (Figure 8A) and at 90% acetonitrile (Figure 8B). Column P (black bars) showed matrix factors significantly less than 1 for all the analytes, indicating ion suppression. In contrast, column A (red bars) showed matrix factors slightly greater than 1 for many of the analytes, indicating ion enhancement. While similar trends were observed at 10% and 90% acetonitrile, more severe suppression for column P and enhancement for column A was observed at 90% acetonitrile. In contrast, at both 10% and 90% acetonitrile, column W (blue bars) showed matrix factors ranging from 0.9 to 1.3, indicating little to no suppression or enhancement from column bleed. We attribute this to the greater hydrolytic stability of the hybrid particle‐based stationary phase for column W.

## 4 CONCLUSIONS

These results show that the silica‐based RP/AX columns (columns A and P) exhibited significant MS signal suppression or enhancement, which appears to be caused by hydrolysis products from the bonded phases and/or the silica particles. In contrast, the hybrid particle‐based column W exhibited minimal MS signal suppression or enhancement caused by column bleed. This is due to the improved hydrolytic stability of the stationary phase for column W, which is a consequence of its ethylene‐bridged hybrid particles and bonded‐phase structure, containing separate C_18_ and AX groups, both attached using stable bonding chemistries. This improved stability makes column W well suited for LC/MS applications, particularly for quantitative methods.

### PEER REVIEW

The peer review history for this article is available at https://publons.com/publon/10.1002/rcm.9098.

## Data Availability

The data that support the findings of this study are available from the corresponding author upon reasonable request.
